# P-695. Analysis of Seasonal Influenza Activity in Los Angeles County throughout the COVID-19 Pandemic

**DOI:** 10.1093/ofid/ofae631.891

**Published:** 2025-01-29

**Authors:** Heidi Ransohoff, Elizabeth Traub, Annabelle de St Maurice

**Affiliations:** Los Angeles Department of Public Health, Los Angeles, California; Los Angeles County Department of Public Health, Los Angeles, CA; University of California Los Angeles, Los Angeles, California

## Abstract

**Background:**

COVID-19 pandemic restrictions impacted flu transmission, leading to lower incidence and high influenza activity outside of the typical respiratory season. Los Angeles County (LAC) Department of Public Health (DPH) typically defines flu season in LAC as the first week of October through the end of March. Because positive flu results became reportable in LAC via electronic laboratory reporting (ELR) in October 2019, this analysis uses this new data source to describe flu positive tests from the current and previous four flu seasons to understand how flu activity in LAC changed throughout the pandemic.

**Figure:**
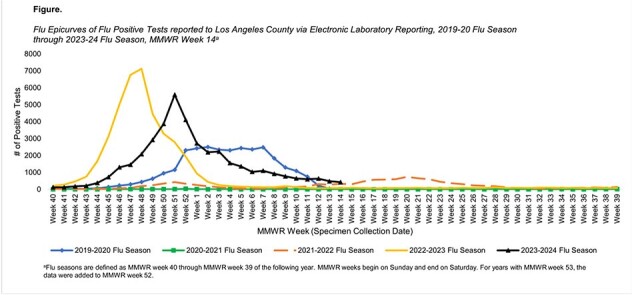

**Methods:**

This analysis includes influenza positive test results for LAC residents reported to DPH via ELR with specimen collection dates from October 2019 to March 2024. Results were deduplicated at the specimen level. Descriptive statistics were used to analyze positive tests.

**Results:**

During the 2023-24 flu season from October 2023 through March 2024, 39,412 positive influenza results were reported (87% flu A; 13% flu B), with the greatest number of positive results for flu A observed in December 2023 and flu B observed in February 2024. During the same months of previous seasons, the number of positive tests ranged from 217 tests in the 2020-21 season to 40,843 tests in the 2022-23 season. Peak months of flu activity ranged from November in the 2022-23 season to May in the 2021-22 season.

For all tests reported during the study period, subtyping was reported for 8% of positive flu A specimens. Lineage typing was reported for 0.04% of positive flu B specimens. During the current flu season, at least 1 positive test has been reported for flu A/H1, flu A/H3, and flu B/Victoria. The last recent positive flu B/Yamagata specimen was reported in January 2020.

**Conclusion:**

Results show that flu activity in LAC during the current flu season appears to be returning to pre-COVID-19 pandemic seasonality; flu B activity peaked later in the season, which has been described for previous flu seasons. During the pandemic, peak months of flu activity varied from what is usually observed. No positive flu B/Yamagata tests since 2020 may support ongoing efforts to assess flu B/Yamagata extinction during the COVID-19 pandemic, prompting the World Health Organization to recommend excluding flu B/Yamagata from flu vaccines.

**Disclosures:**

**All Authors**: No reported disclosures

